# Pseudotime estimation: deconfounding single cell time series

**DOI:** 10.1093/bioinformatics/btw372

**Published:** 2016-06-17

**Authors:** John E. Reid, Lorenz Wernisch

**Affiliations:** MRC Biostatistics Unit, Cambridge CB2 0SR, UK

## Abstract

**Motivation:** Repeated cross-sectional time series single cell data confound several sources of variation, with contributions from measurement noise, stochastic cell-to-cell variation and cell progression at different rates. Time series from single cell assays are particularly susceptible to confounding as the measurements are not averaged over populations of cells. When several genes are assayed in parallel these effects can be estimated and corrected for under certain smoothness assumptions on cell progression.

**Results:** We present a principled probabilistic model with a Bayesian inference scheme to analyse such data. We demonstrate our method’s utility on public microarray, nCounter and RNA-seq datasets from three organisms. Our method almost perfectly recovers withheld capture times in an *Arabidopsis* dataset, it accurately estimates cell cycle peak times in a human prostate cancer cell line and it correctly identifies two precocious cells in a study of paracrine signalling in mouse dendritic cells. Furthermore, our method compares favourably with Monocle, a state-of-the-art technique. We also show using held-out data that uncertainty in the temporal dimension is a common confounder and should be accounted for in analyses of repeated cross-sectional time series.

**Availability and Implementation:** Our method is available on CRAN in the DeLorean package.

**Contact:** john.reid@mrc-bsu.cam.ac.uk

**Supplementary information:** Supplementary data are available at *Bioinformatics* online.

## 1 Introduction

Many biological systems involve transitions between cellular states characterized by gene expression signatures. These systems are typically studied by assaying gene expression over a time course to investigate which genes regulate the transitions. An ideal study of such a system would track individual cells through the transitions between states. Studies of this form are termed *longitudinal*. However, current medium and high-throughput assays used to measure gene expression destroy cells as part of the protocol. This results in *repeated cross-sectional* data wherein each sample is taken from a different cell.

This study analyses the problem of variation in the temporal dimension: cells do not necessarily transition at a common rate between states. Even if several cells about to undergo a transition are synchronized by an external signal, when samples are taken at a later time point each cell may have reached a different point in the transition. This suggests a notion of pseudotime to model these systems. Pseudotime is a latent (unobserved) dimension which measures the cells’ progress through the transition. Pseudotime is related to but not necessarily the same as laboratory capture time.

Variation in the temporal dimension is a particular problem in repeated cross-sectional studies as each sample must be assigned a pseudotime individually. In longitudinal studies, information can be shared across measurements from the same cell at different times.

Inconsistency in the experimental protocol is another source of variation in the temporal dimension. It may not be physically possible to assay several cells at precisely the same time point. This leads naturally to the idea that the cells should be ordered by the pseudotime they were assayed.

The exploration of cell-to-cell heterogeneity of expression levels has recently been made possible by single cell assays. Many authors have investigated various biological systems using medium-throughput technologies such as qPCR (Buganim *et al.*, 2012; Chung *et al.*, 2014; Guo *et al.*, 2010; Kouno *et al.*, 2013) and nCounter (McDavid *et al.*, 2014; Yosef *et al.*, 2013) or high-throughput technologies such as RNA-seq (Brennecke *et al.*, 2013; Islam *et al.*, 2011; Pollen *et al.*, 2014; Shalek *et al.*, 2013, 2014; Tang *et al.*, 2010; Trapnell *et al.*, 2014; Treutlein *et al.*, 2014). These studies have shown that cellular heterogeneity is prevalent in many organisms and regulatory systems. The variation in gene expression underlying this cellular heterogeneity has been attributed to several causes both technical and biological (Brennecke *et al.*, 2013; Islam *et al.*, 2011; Kouno *et al.*, 2013; Shalek *et al.*, 2013). While accounting for variation in expression levels, none of these studies investigated how much is attributable to uncertainty in the temporal dimension. Conversely, methods such as Monocle and Wanderlust (described below) have attempted to place cells in a pseudotemporal ordering but do not explicitly model variation in the data.

Analyses of medium and high-throughput expression assays often use dimension reduction techniques. Anywhere between 40 and several tens of thousands of gene expression levels may have been measured in each sample. This high-dimensional data can often be better analysed after projection into a low (two or three) dimensional latent space. Often this projection results in a natural clustering of cells from different time points or of different cell types which can then be related to the biology of the system. Such clusterings may suggest hypotheses about likely transitions between clusters and their relationship in time.

Dimension reduction has a large literature and there are many available methods. Here, we give a few examples of some that have been used in single cell expression analyses.

Principal components analysis (PCA) is prevalent in analyses of expression data (Islam *et al.* 2011; Pollen *et al.* 2014; Shalek *et al.* 2014; Tang *et al.* 2010). PCA finds linear transformations of the data that preserve as much of the variance as possible. In one example typical of single cell transcriptomics, Guo *et al.* (2010) studied the development of the mouse blastocyst from the one-cell stage to the 64-cell stage. They projected their 48-dimensional qPCR data into two dimensions using PCA. Projection into these two dimensions clearly separated the three cell types present in the 64-cell stage.

Multi-dimensional scaling (MDS) is another popular dimension reduction technique. MDS aims to place each sample in a lower dimensional space such that distances between samples are conserved as much as possible. Kouno *et al.* (2013) used MDS to study the differentiation of THP-1 human myeloid monocytic leukemia cells into macrophages after stimulation with PMA. Their primary MDS axis explained the temporal progression through the differentiation, their secondary MDS axis explained the early-response of the cells to the stimulation they had undergone.

Independent components analysis (ICA) projects high dimensional data into a latent space that maximizes the statistical independence of the projected axes. Trapnell *et al.* (2014) used ICA to investigate the differentiation of primary human myoblasts. The latent space serves as a first stage in their pseudotime estimation algorithm Monocle (see below).

Gaussian process (GP) latent variable models (GPLVMs) are a dimension reduction technique related to PCA. They can be seen as a non-linear extension (Lawrence, 2005) to a probabilistic interpretation of PCA (Tipping and Bishop, 1999). Buettner *et al.* (2014) and Buettner and Theis (2012) used GPLVMs to study the differentiation of cells in the mouse blastocyst. They used qPCR data from Guo *et al.* (2010) who had analysed the expression of 48 genes in cells spanning the 1- to 64-cell stages of blastocyst development. Buettner *et al.* were able to uncover subpopulations of cells at the 16-cell stage, one stage earlier than Guo *et al.* had identified using PCA.

The latent space in all of the methods above is unstructured: there is no direct physical or biological interpretation of the space and the methods do not directly relate experimental covariates such as cell type or capture time to the space. The samples are placed in the space only to maximize some relevant statistic, although the analysis often reveals some additional structure. For example, one axis may coincide with the temporal dimension of the data, or cell types may be clearly separated. In these cases, the structure has been inferred in an unsupervised manner. However, there is no guarantee that the methods above will uncover any specific structure of interest, for example, a pseudotime ordering.

Here, we propose to impose an a priori structure on the latent space. In the model presented in this article, the latent space is one-dimensional and the structure we impose on the space relates it to the temporal information of the cell capture times. That is the latent space represents the pseudotime.

A number of methods have been proposed to estimate pseudotimes in gene expression time series. Äijö *et al.* (2014) proposed a temporal scaling method DyNB to estimate pseudotimes. DyNB shifts the observed time by a multiplicative factor representing speed of transition through the process. It is applicable to longitudinal rather than repeated cross-sectional time series. Äijö *et al.* modelled RNA-seq count data from human Th17 cell differentiation using a negative binomial distribution with a time-varying mean. For each of three biological replicates, they analysed subpopulations of cells at given time points resulting in three time series of longitudinal data. The time-varying mean was fit using a GP over the scaled pseudotime space. They compared this pseudotime based model favourably with a similar model that only used the capture time points.

Trapnell *et al.* (2014) have developed the Monocle pseudotime estimation algorithm. Monocle is a two-stage procedure: first, it uses the ICA dimension reduction algorithm to map gene expression data into a low-dimensional space; second, it finds the minimal spanning tree (MST) over the samples’ locations in this space. This spanning tree is used to assign a pseudotime to each cell. Trapnell *et al.* show how Monocle can be used to identify pseudotemporal ordering, switch-like changes in expression, novel regulatory factors and sequential waves of gene regulation.

Shin *et al.* (2015) have developed the Waterfall pseudotime estimation algorithm that is closely related to Monocle. Waterfall reduces the dimension of the data to 2 using PCA, uses k-means clustering in this space to group the data and calculates a MST over the cluster centroids to induce a pseudotime trajectory. After estimating the pseudotime, Waterfall uses a hidden Markov model (HMM) to infer when genes switch on and off.

Campbell *et al.* have a body of work investigating pseudotime estimation. They developed the Embeddr R package (Campbell *et al.*, 2015) that uses a similar approach to Monocle but with some significant differences. They use Laplacian eigenmaps for dimensionality reduction instead of ICA and fit pseudotime trajectories using principal curves rather than MSTs. Dissatisfied with the point estimates of pseudotimes such an approach generates they subsequently developed a fully Bayesian probabilistic model using GPs (Campbell and Yau, 2015).

Wanderlust is a graph-based pseudotime estimation algorithm developed by Bendall *et al.* (2014). Wanderlust arranges the high-dimensional input data into a nearest neighbour graph wherein cells that have similar expression profiles are connected. Wanderlust then applies a repetitive randomized shortest path algorithm to assign an average pseudotime to each cell. Bendall *et al.* used Wanderlust to analyse human B cell lymphopoiesis.

The Monocle, Waterfall, Embeddr and Wanderlust algorithms do not make a connection between the cell capture times and the estimated pseudotime explicitly. This has two effects: first, in the inference of the pseudotime, nonsensical results are possible such as pseudotimes whose order is far from the capture times; second, the estimated pseudotimes are not on the same scale as the capture times, they are quantified in arbitrary temporal units.

Oscope (Leng *et al.*, 2015) is a method for detecting groups of oscillatory genes. When a group is detected Oscope uses an extended nearest insertion algorithm to place the cells in pseudotime order.

## 2 Approach

Gaussian processes are Bayesian models that are well suited to model expression profiles and capture the uncertainty inherent in noisy data. Bayesian inference in GPs can be performed analytically and provides posterior mean estimates with a full covariance structure. A GP is parameterized by a mean and a covariance function. For more details, Rasmussen and Williams (2006) have published a comprehensive review.

GPs have been used extensively to model time series and other phenomena in biological systems: Stegle *et al.* (2010) designed a two-sample test for differential expression between time series using GPs; Honkela *et al.* (2010) used GPs to model expression profiles of transcription factors in an ODE based model of gene regulation; Äijö and Lähdesmäki (2009) used GP models of regulatory functions to infer gene networks and Kirk *et al.* (2012) used GPs to model time series in a multiple dataset integration method.

## 3 Methods

### 3.1 Data

Our method has been designed to analyse single cell data but there is no technical reason why each sample must be from a cell. The model itself and notion of pseudotime would suit many repeated cross-sectional datasets. Indeed one of the datasets used in our results section is from whole leaf microarray assays. However, for consistency of explanation, we refer to each sample as a cell in this article.

Our method works on data with a simple structure. First, it expects gene expression data on a logarithmic scale, such as Ct values from qPCR experiments or log transformed counts from RNA-seq experiments. Second, it requires a *capture time* for each cell. This specifies at which time point that cell was sampled. We assume the data has already been adjusted for cell size (see Supplementary Materials).

Our notation for the data is: *G* is the number of genes assayed; *C* is the number of cells sampled; $x_{g,c}$ is the expression level of gene *g* in cell *c* where 1≤g≤G and 1≤c≤C; the capture time of cell *c* is *k_c_* where $k_{c}\in \{\kappa_{1},\kappa_{2},\ldots ,\kappa_{T}\}$ and *T* is the number of distinct capture times.

### 3.2 Model

The primary latent variables in our model are the pseudotimes. The model assigns a pseudotime to each cell such that the induced gene expression profiles over the latent pseudotime space have low noise levels and are smooth.

Our model captures several aspects of the data: first, the data are noisy which we model in a gene-specific fashion; second, we expect the expression profiles to be smooth; third, we expect the pseudotime of each cell not to stray too far from its capture time.

The model can be split into several parts: one part represents the gene expression profiles; another part represents the pseudotimes associated with each cell and another part links the expression data to the profiles.

### 3.3 Gene expression profiles

The expression profiles are modelled using GPs. The expression profile *y_g_* of gene *g* is a draw from a GP

(1) $$y_{g}∼GP(\phi_{g},\Sigma_{g})$$

where $\phi_{g}$ is a (constant) gene-specific mean function estimated from the data and Σ_*g*_ is a gene-specific covariance function. The expression profiles are functions of pseudotime and as such the covariance function relates two pseudotimes.

(2) $$\Sigma_{g}(\tau_{1},\tau_{2})=\psi_{g}\Sigma_{\tau }(\tau_{1},\tau_{2})+\omega_{g}\delta_{\tau_{1},\tau_{2}}$$

Here, $\Sigma_{\tau }$ is a covariance function that defines the covariance structure over the pseudotimes. $\Sigma_{\tau }$ imposes the smoothness constraints that are shared across genes; *ψ_g_* parameterizes the amount of temporal variation this gene profile has and *ω_g_* models the noise levels for this gene. Log-normal priors for the *ψ_g_* and *ω_g_* are parameterized as

(3) $$\log\psi_{g}∼N(\mu_{\psi },\sigma_{\psi })$$

(4) $$\log\omega_{g}∼N(\mu_{\omega },\sigma_{\omega })$$

For data with many cells, we use a sparse GP approximation (Snelson and Ghahramani, 2006) which has a computational complexity of $O(GCM^{2})$ where M≪C is a parameter of the approximation. This is a significant improvement on the $O(GC^{3})$ complexity of the exact model. The details of the approximation are in the Supplementary Materials as are running times for the results in this article.

### 3.4 Pseudotimes

The pseudotime *τ_c_* for cell *c* is given a prior centred on the time the cell was captured. We use a normal prior as it reflects our beliefs well. There are no conjugacy issues in our inference scheme and it would be straightforward to use any prior distribution.

(5) $$\tau_{c}∼N(k_{c},\sigma_{\tau })$$

Each *τ_c_* is used in the calculation of the covariance structure over pseudotimes $\Sigma_{\tau }$. $\Sigma_{\tau }$ is taken to be a Matern_3∕2_ covariance function. Our experience shows that this function captures our smoothness constraints well although any reasonable covariance function could be used.

(6) $$\Sigma_{\tau }(\tau_{1},\tau_{2})={\text{Matern}}_{3/2}(r=\frac{\left|\tau_{1}-\tau_{2}\right|}{l})=(1+\sqrt{3}r)\exp[-\sqrt{3}r]$$

where *l* is a length-scale hyperparameter shared across the genes.

For cyclic data such as from the cell cycle or circadian rhythms, we expect the expression profiles to be periodic. We can model this explicitly by a transformation of *r* in Equation (6). We replace *r* by $r_{\Omega }$

(7) $$r_{\Omega }=\frac{\Omega }{2}\sin\frac{\pi r}{\Omega }$$

This has the effect of restricting the GP prior to periodic functions with period Ω.

### 3.5 Expression data

The model links the expression data to the expression profiles by evaluating the profiles at the pseudotimes.

(8) $$x\prime_{g,c}=y_{g}(\tau_{c})$$

### 3.6 Relationship to other models

Briefly, our model can be interpreted as a one-dimensional GPLVM with a prior structure on the latent pseudotime space. The GPLVM model is a non-linear version of probabilistic PCA. In probabilistic PCA, the locations of the data in the latent space are given a Gaussian prior with zero mean and unit covariance. In our model, the analogous latent variables are the pseudotimes. Our model gives the pseudotimes a structured prior rather than a standard normal: that is, we relate the latent pseudotimes to the capture times of the cells using a Gaussian prior.

### 3.7 Inference

All of the hyperparameters $\mu_{\psi },\sigma_{\psi },\mu_{\omega },\sigma_{\omega }$ are estimated by an empirical Bayes procedure (see Supplementary Materials). The hyperparameters $l,\sigma_{\tau }$ are supplied directly by the user of our method.

As with many hierarchical models, the parameters can have several posterior modes. For instance, much of the variation in typical single cell assay data could be explained by smooth expression profiles with high noise levels. Alternatively, the same data could also be explained by rough expression profiles with low noise levels. Our model aims to balance these conflicting explanations and find parameters to fit the data with reasonable noise levels and expression profiles that are neither too smooth nor too rough. Selecting suitable hyperparameters for the parameter priors is important to avoid unrealistic regions of parameter space. We have found an empirical Bayes approach useful in this regard (see Supplementary Materials).

Our model is coded using the Stan probabilistic modelling language (Carpenter,B., *et al*., 2016.). The Stan package provides various inference algorithms. In this work, we have used the No-U-Turn Hamiltonian Markov chain Monte Carlo sampler (NUTS) (Hoffman and Gelman, 2014) and the ADVI variational Bayes algorithm (Kucukelbir *et al.*, 2015). In theory using the NUTS sampler gives us samples from the full posterior of the model. However, the model is multimodal with respect to the pseudotime assignments and sometimes this makes it difficult for the sampler to mix samples from the full posterior. The multimodality occurs as there may be many pseudotemporal orderings of the cells that give smooth expression profiles. Moving between these modes is difficult for the sampler since in order to change the order of cells they must pass each other in pseudotime. If the cells’ expression profiles are sufficiently different the likelihood of the sampler passing this configuration can be very low. In these cases, the sampler may only visit a few modes of the posterior. This difficulty in mixing is not unique to our model. Many other models such as k-means clustering exhibit similar behaviour. In these models, it is common practice to use a single sample as a point estimate of the latent variables. Typically, the sample with the highest probability under the model is selected. The Stan NUTS sampler provides $\hat{R}$ statistics that give confidence in the mixing over pseudotime (Brooks and Gelman, 1998). These statistics can be evaluated on a dataset-by-dataset basis and a point estimate or the full posterior can be used for further analysis. The NUTS sampler is slower than the approximate inference provided by the ADVI algorithm which we use when the problem size is large.

In order to further mitigate the pseudotime mixing problem, we use naive heuristics to initialize our MCMC chains and ADVI starting points (see Supplementary Materials).

### 3.8 Validation methods

In the results section, we analyse specific datasets and validate the inferences from our model in several biological contexts. However, we also wished to validate our model technically. We base this technical validation on the smoothness of expression profiles induced on held-out genes. The held-out genes are not used during model fitting and are only used in the validation stage. To evaluate the smoothness, we developed a basic statistic to capture this concept. Given expression values $x\prime_{g,c}$ for a held-out gene *g* over cells 1≤c≤C, pseudotimes $\tau_{1},\ldots ,\tau_{C}$ and an ordering $z_{1},\ldots ,z_{C}$ such that

$$\tau_{z_{1}}\leq \ldots \leq \tau_{z_{C}}$$

we define the roughness of the gene in terms of the differences of consecutive expression measurements under the ordering given by the pseudotimes

(9) $$R_{g}(z)=\frac{1}{\sigma_{g}}\sqrt{\frac{1}{C-1}\sum_{c=1}^{C-1}{\left(x\prime_{g,z_{c}}-{x^{\prime }}_{g,z_{c+1}}\right)}^{2}}$$

where *σ_g_* is the standard deviation of the expression measurements. Clearly, low *R_g_* values should correlate with smooth profiles and high *R_g_* values should correlate with rough profiles.

One benefit of defining *R_g_* in terms of the pseudotime ordering rather than the pseudotime itself is that it is easy to generate random orderings under a suitable null hypothesis. The null hypothesis we use is that the cells are ordered by capture time but within a capture time are equally likely to have any order. That is, we generate random orderings that respect the capture times. We use a one-sided *t*-test to determine if the mean of the roughness of the pseudotime orderings is less than the mean of the roughness of orderings drawn under the null hypothesis. Defining *R_g_* in terms of the ordering rather than the actual pseudotime also allows us to use it to compare the roughness of orderings from other methods such as Monocle.

## 4 Results and discussion

We used our model to analyse three sets of data from three different organisms assayed using three different technologies: whole leaf *Arabidopsis thaliana* microarrays (Windram *et al.*, 2012); nCounter single cell profiling of a human prostate cancer cell line (McDavid *et al.*, 2014) and single cell RNA-seq of mouse dendritic cells (Shalek *et al.*, 2014).

Windram *et al.* (2012) examined the response of *Arabidopsis thaliana* to infection by the necrotrophic fungal pathogen *Botrytis cinerea*. They generated high-resolution time series over 48 h for an infected condition and a control condition. We investigated if our model could estimate the correct order for the samples if their exact capture times were withheld.

### 4.1 The model correctly estimates withheld sample times

Windram *et al.* (2012) measured expression levels every 2 h resulting in 24 distinct capture time points. We grouped these 24 time points into four low-resolution groups, each consisting of six consecutive time points. We then asked our model to estimate the pseudotimes associated with each sample but only provided it with the low-resolution group labels. We fit 100 of the 150 genes mentioned in the text of Windram *et al.*’s publication. We used the remaining 50 genes as held-out data to validate the fit.

**Fig. 1.**
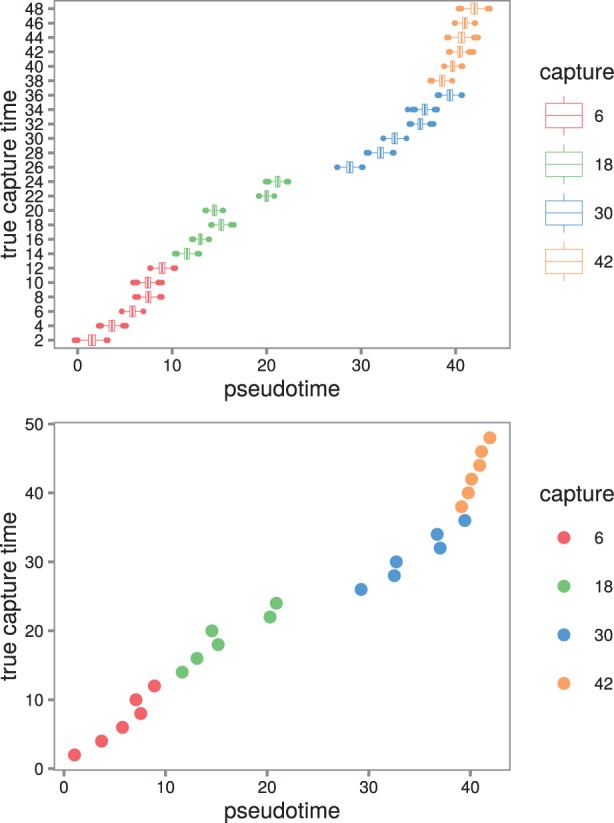
Pseudotime estimates for the samples from the Windram *et al.*’t (2012) *Arabidopsis* data. (**Top**) Boxplots of the full pseudotime posteriors. The estimated pseudotimes are in good agreement with the true capture times. The model tends to spread the samples out around the 20-h mark in pseudotime. Presumably the expression profiles vary the most at this point. In addition, the samples are spread out more broadly in pseudotime (between -20 and 60 h) compared to the true capture times. (**Bottom**) The pseudotimes estimated by the best sample from the posterior plotted against the true capture times

We used the ADVI variational Bayes algorithm to estimate pseudotimes for each sample in the infected condition (see Fig. 1). The profiles induced by the inferred pseudotimes were smooth (see Supplementary Materials).

**Fig. 2.**
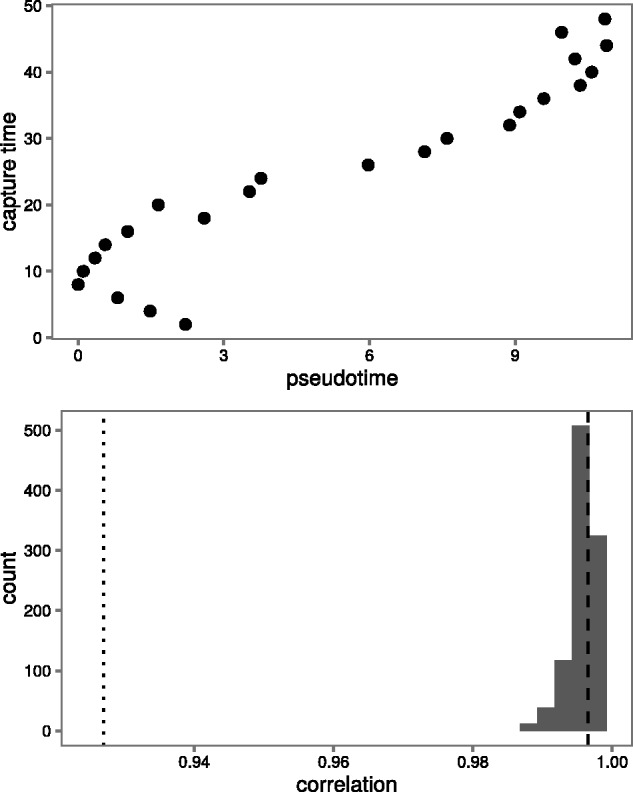
A comparison of the performance of our method and the Monocle algorithm. (**Top**) Pseudotimes predicted by the Monocle algorithm (ρ=0.927). (**Bottom**) Posterior of the Spearman correlation between estimated pseudotimes from our model and true capture times. The Spearman correlation of the Monocle pseudotimes with the true capture times is shown as a dotted line. The Spearman correlation of the best sample with the true capture times is shown as a dashed line

The Spearman correlation between estimated pseudotimes from the posterior and the true capture times was high (posterior mean ρ=.996) (see Fig. 2 top left). The correlation for the best posterior sample had Spearman correlation ρ=.996.

### 4.2 Our model fits the data better than Monocle

We also used the Monocle algorithm to predict pseudotimes for the same 100 genes (see Supplementary Materials). Monocle was unable to recover the capture times for cells from the first low resolution group (see Fig. 2). The Spearman correlation between Monocle’s estimated pseudotimes and the true capture times was not as high (ρ=0.927) as that for our estimates. Monocle’s difficulty in resolving the correct ordering can be explained by its inability to use prior information that could resolve the first two groups of cells.

### 4.3 The pseudotimes induce smooth profiles on held-out genes

We calculated roughness statistics *R_g_* (see Section 3) for the 50 genes that we had not used to fit the model and averaged over genes. We did the same for 1000 pseudotime orderings sampled under the null hypothesis. The posterior mean of the *R_g_* of the pseudotimes estimated by our model were significantly smaller than those from the null hypothesis ($p<10^{-15}$ one-sided *t*-test). We calculated the roughness statistic for the pseudotime ordering estimated by Monocle. This was significantly higher than the roughnesses in our posterior (see Supplementary Materials).

McDavid *et al.* (2014) were interested in the effect of the cell cycle on the single cell gene expression. They assessed this effect by assaying the expression levels of 333 genes in 930 cells across three human cell lines using nCounter single cell profiling (Geiss *et al.*, 2008). Based on these data, they concluded that the cell cycle explains just 5–17% of expression variability.

CycleBase (Santos *et al.*, 2015) is a database of cell cycle related genes and time series expression data. It contains metadata including the time in the cell cycle at which expression peaks for cell cycle related genes. To evaluate our model, we assessed how closely the peaks in the expression profiles estimated by our model from McDavid *et al.*’s (2014) data matched the CycleBase peak times. Additionally, as a baseline, we compared peaks estimated from the raw expression data by a naive algorithm to the Cyclebase peak times. We also used Oscope to estimate pseudotimes (see Supplementary Materials) and compared the peaks to the CycleBase peaks.

### 4.4 The model recovers cell cycle peak times

**Fig. 3.**
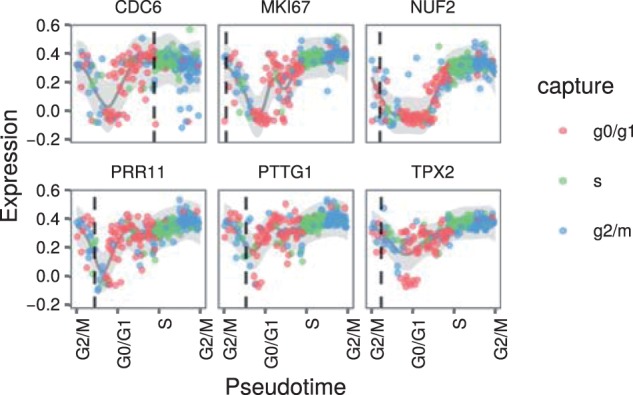
Expression profiles over pseudotime from the McDavid *et al.* (2014) cell cycle data. The pseudotimes are those from the best sample. Note the circular *x* axis: the first and last labels are both for the G2/M stage. The genes were selected based on high ratios of temporal variance to noise. Each point represents the expression of the given gene in a cell. The points are coloured by the cell cycle stage with which the cell was labelled by McDavid *et al.* The dark grey line represents the posterior mean of the expression profile for the gene and the shaded grey ribbon represents two standard deviations either side of this mean. The vertical dotted lines are the peak times as defined by the CycleBase database

We used ADVI to fit our model to the 361 cells from the PC3 human prostate cancer cell line and chose the top 56 differentially expressed genes according to McDavid *et al.*’s (2014) differential expression test. We mapped cells identified by McDavid *et al.* (2014) as G0/G1, S and G2/M to capture times of 1, 2 and 3, respectively. We used a length scale of 5 and set $\sigma_{\tau }=\frac{1}{2}$. To model the cyclic nature of the cell cycle, we used a periodic covariance function with period Ω = 3. This ensured the expression profiles were periodic and transitions between all the cell cycle phases were consistent. We show expression profiles from the best sample in Figure 3.

In order to test the fit of our model, we estimated peak times from the expression profiles fit by the model and compared these to peak times as defined by the CycleBase database. To quantify this fit, we calculated the root mean squared error (RMSE) between the CycleBase defined peak times and our estimates (RMSE = 14.5).

We wished to understand how well our model estimated peak times compared to naive estimates. We made naive estimates from the raw expression data as follows. Each cell in McDavid *et al.*’s (2014) data had been labelled with one of the cell cycle phases. We identified the cell with maximal raw expression value for each gene. The middle of the cell cycle phase with which this cell was labelled was used as the naive estimate of the gene’s peak time. These estimated peak times had a RMSE of 22.4 which is 54% larger than the RMSE of our estimated peak times. This demonstrates that our model’s expression profiles capture information present in the data at a higher temporal resolution than the raw labels. We also used Oscope to estimate pseudotimes for the cells (see Supplementary Materials). The RMSE associated with these estimates was 33.3%.

Shalek *et al.* (2014) generated repeated cross-sectional time courses of the response of primary mouse bone-marrow-derived dendritic cells in three separate conditions using single-cell RNA-seq. We analysed the data on the lipopolysaccharide stimulated (LPS) condition using our model.

### 4.5 The model identifies precocious cells

Shalek *et al.* (2014) identified a core antiviral module of genes that are expressed in conditions such as LPS after 2–4 h. They also identified two cells captured at 1 h that had this module switched on precociously. Other cells captured at 1 h did not express the genes in this module. This concept that some cells can progress through pseudotime faster than others is exactly the concept that our model is designed to capture. We were interested to establish if our model could place these cells at later pseudotimes than other cells captured at 1 h.[AQ7]

We used ADVI to fit our sparse model to 307 cells from the LPS condition including the two precocious cells captured at 1 h. Shalek *et al.* (2014) defined several gene modules in their publication that show different temporal patterns of expression across the LPS time course. We selected the 74 genes from the clusters Id, IIIb, IIIc, IIId with the highest temporal variance relative to their noise levels. We set $\sigma_{\tau }=1$ and used a length scale of 5.

**Fig. 4.**
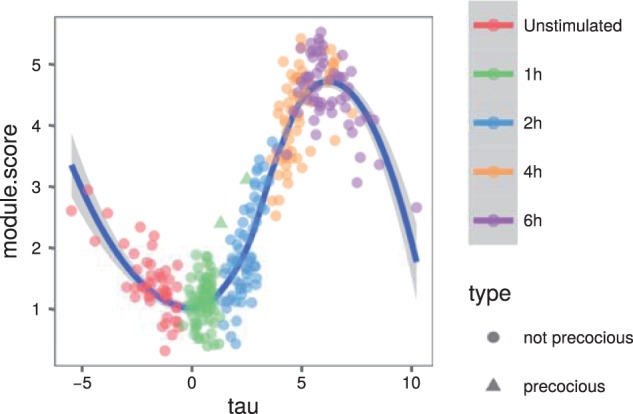
The module score (as defined by Shalek *et al.*) of core antiviral genes over pseudotime. The two precocious cells captured at 1 h are plotted as triangles. These two cells have been placed at a later pseudotime than the other cells captured at 1 h. A Loess curve has also been plotted through the data

Figure 4 shows the module scores of the core antiviral genes (as defined by Shalek *et al.* (2014)) over pseudotime. The two precocious cells have been fit with a pseudotime in the middle of the 2-h capture cells. We note that sometimes our model can best fit outlying cells by pushing them to the extremes of pseudotime. For this reason, we do not necessarily trust the Loess curve estimates of the module score at these extreme pseudotimes.

### 4.6 The model identifies smooth expression profiles

We calculated roughness statistics *R_g_* (see Section 3) for 100 genes that we had not used to fit the model and averaged over genes. We did the same for 1000 pseudotime orderings sampled under the null hypothesis. The posterior mean of the *R_g_* of the pseudotimes estimated by our model were significantly smaller than those from the null hypothesis ($p<10^{-15}$ one-sided *t*-test).

## 5 Conclusion

We have presented a principled probabilistic model that accounts for uncertainty in the capture times of repeated cross-sectional time series. We have fit our model to three separate datasets each using a different biological assay (microarrays, single cell nCounter and single cell RNA-seq) in three organisms (human, mouse and *Arabidopsis*). Our model provided plausible estimates of pseudotimes on all the datasets. We validated these estimates technically by evaluating the smoothness of the expression profiles of held-out genes in two of the datasets. These profiles are significantly smoother than expected under the null model. In addition, we validated the estimates biologically using obfuscated capture times (in the *Arabidopsis* dataset), data from separate experiments (cell cycle peak times) and independent analyses (identification of precocious cells). Overall these results demonstrate that uncertainty in the temporal dimension should not be ignored in repeated cross-sectional time series of single cell data and that our method captures and corrects for these effects.

Our method has a number of attractive attributes. It explicitly estimates pseudotimes in contrast to methods such as Monocle and Wanderlust which estimate orderings of cells. The pseudotimes are on the same scale as the experimental capture times. The orderings estimated by Monocle and Wanderlust have no scale. In our model, consecutive cells that have diverse expression profiles are placed further apart in pseudotime than similar cells. Thus, our pseudotime estimates quantify the rate of change of the system. For example, in the *Arabidopsis* example, we analysed the cells are spread out in pseudotime around the 20-h mark (Fig. 1) suggesting changes in expression levels in response to the infection are greatest at this time point.

Our method uses GPs which are a natural framework to model noisy expression profiles. GPs are well established probabilistic models for time series. They provide more than just point estimates of the profiles, they also provide a measure of posterior uncertainty. This is useful in downstream analyses such as regulatory network inference. A GP model is characterized by its covariance function and associated parameters and the covariance functions in our model have interpretable parameters: gene-specific temporal variation and noise. We have also demonstrated how a GP framework is suitable for modelling periodic expression profiles such as cell cycle expression profiles. The primary limitation of GPs for our model is that inference complexity scales cubically in the number of samples. For this reason, our method is not applicable to data from many hundreds or thousands of cells like Monocle and Wanderlust.

Inference in our model is performed using Markov chain Monte Carlo. This technique provides a full posterior distribution over the model parameters. However, mixing over the pseudotime parameters in our model can be difficult and we found that our model did not mix well when fit to the cell cycle dataset. In this case, we analysed expression profiles from the sample with highest log probability and found they estimated cell cycle peak times well.

Single cell assays give us an exciting opportunity to explore heterogeneity in populations of cells. As the technology develops and the cost of undertaking such assays drops, they are destined to become commonplace. In addition, high-throughput longitudinal studies remain impractical and for the foreseeable future the majority of such time series will be repeated cross-sectional in nature. Until this changes, there will be challenges associated with estimating uncertainty in the capture times and variation in the rate of progress of individual cells through a system. Our method explicitly models these effects and is a practical tool for analysis of such repeated cross-sectional time series. Furthermore, in contrast to Wanderlust, our method only depends on open-source software and is available under a liberal open-source license.

## Supplementary Material

Supplementary Data
