# Supplementary material for: Pseudotime estimation: deconfounding single cell time series
Source: Bioinformatics. 2016 Jun 17;32(19):2973–80. doi: 10.1093/bioinformatics/btw372 (PMC5039927; doi:10.1093/bioinformatics/btw372)
Supplement: Supplementary Data [file supp_32_19_2973__index.html]

Pseudotime estimation: deconfounding single cell time series — Supplementary Data 

# Pseudotime estimation: deconfounding single cell time series

## Supplementary Data

files

- Supplementary Data - pdf file
