# Supplementary material for: Pseudotime estimation: deconfounding single cell time series
Source: Bioinformatics. 2016 Jun 17;32(19):2973–80. doi: 10.1093/bioinformatics/btw372 (PMC5039927; doi:10.1093/bioinformatics/btw372)
Supplement: Supplementary Data [file supp_btw372_suppl-mat.pdf]

Methods

Cell size adjustment

Single cell expression measurements can often contain per-cell biases that present as differences in basal levels of transcription. These can be caused by biological effects such as cell size or technical effects such as lysis efficiency or sequencing depth. We use a technique based on the method proposed by [1] to account for these effects.

Given a subset of the genes $\mathcal{G} \subseteq \{1, \dots, G\}$ and a subset of the cells $\mathcal{C} \subseteq \{1, \dots, C\}$ we define the cell size for cell $c \in \mathcal{C}$ as

$$S_c(\mathcal{G}, \mathcal{C}) = \text{Median}_{g \in \mathcal{G}}\{x_{g,c} - \text{Mean}_{c \in \mathcal{C}}\{x_{g,c}\}\} \tag{1}$$

The median is used as it provides a robust estimate of the differences across genes. We estimate the cell sizes separately for the cells grouped by capture time,

$$\mathcal{C}_t = \{c : k_c = \kappa_t\}$$

only using those genes that are expressed in at least half of the cells,

$$\mathcal{G}_t = \{g : x_{g,c} > 0 \text{ for at least half of } c \in \mathcal{C}_t\}$$

and apply the cell size as a correction to the raw data

$$x'_{g,c} = x_{g,c} - S_c(\mathcal{G}_t, \mathcal{C}_t) \tag{2}$$

where t is such that $k_c = \kappa_t$. In all that follows we use $x'_{g,c}$ rather than $x_{g,c}$ as our expression data.

Sparse Gaussian process approximation

We use Snelson and Ghahramani's sparse Gaussian process with pseudoinputs approximation [3]. We choose a set of M pseudoinputs, u evenly spaced in the range $[\kappa_{\min} - 3\sigma_\tau, \kappa_{\max} + 3\sigma_\tau]$ where $\kappa_{\min}, \kappa_{\max}$ are the smallest and largest capture times. Following Quiñonero and Rasmussen's notation [2] for Snelson and Ghahramani's Sparse Gaussian Processes using pseudoinputs, we have the approximation

$$\mathcal{N}(\phi_g, \Sigma_g) \approx \mathcal{N}(\phi_g, \psi_g(Q_{\tau,\tau} + \text{diag}[\Sigma_\tau - Q_{\tau,\tau}]) + \omega_g I)$$

where $Q_{\tau,\tau} = \Sigma_{\tau,u}\Sigma_{u,u}^{-1}\Sigma_{u,\tau}$. Note that Quiñonero and Rasmussen call this the Fully Independent Training Conditional (FITC) approximation.

Variance of draw from multivariate Gaussian

Suppose we have a zero-mean Gaussian

$$\mathbf{x} = (x_1, \dots, x_D) \sim \mathcal{N}(0, K)$$

we wish to estimate the expected sample variance

$$V(\mathbf{x}) = \frac{1}{D}\sum_d (x_d - \bar{x})^2 \qquad \text{where} \quad \bar{x} = \frac{1}{D}\sum_d x_d$$

and all sums are from 1 to D. Setting $V_d(\mathbf{x}) = (x_d - \bar{x})^2$ we have

$$V(\mathbf{x}) = \frac{1}{D}\sum_d V_d(\mathbf{x})$$

and

$$\langle V_d(\mathbf{x})\rangle = \langle x_d^2\rangle - 2\langle x_d \bar{x}\rangle + \langle \bar{x}^2\rangle$$

but

$$\begin{aligned}
\langle x_d^2\rangle &= K_{d,d} \\
\langle x_d \bar{x}\rangle &= \frac{K_{d,.}}{D} \\
\langle \bar{x}^2\rangle &= \frac{1}{D}\sum_d \langle x_d \bar{x}\rangle = \frac{K_{.,.}}{D^2}
\end{aligned}$$

where a dot represents summation over that index, so

$$\begin{aligned}
\langle V(\mathbf{x})\rangle &= \frac{1}{D}\left[\sum_d K_{d,d} - \frac{1}{D}K_{.,.}\right] \\
&= \frac{1}{D}\sum_d K_{d,d} - \frac{1}{D^2}K_{.,.} \\
&= \text{Mean}\{\text{Diag}(K)\} - \text{Mean}\{K\}
\end{aligned}$$

Hyperparameter estimation

We need to estimate how much of the variation in the data is due to noise and how much is due to temporal variation in the underlying expression profiles. We use a simple approach that might slightly overestimate both sources of variation but works well enough in our experience. First we group the expression measurements by gene and capture time to calculate means and variances.

$$\mathbb{M}_{g,t} = \text{Mean}_c\{\hat{x}_{g,c} : k_c = \kappa_t\} \tag{3}$$

$$\mathbb{V}_{g,t} = \text{Var}_c\{\hat{x}_{g,c} : k_c = \kappa_t\} \tag{4}$$

We estimate the gene-specific noise levels by assuming that all the within-time variation in the data is due to noise

$$\hat{\omega}_g = \text{Mean}_t\{\mathbb{V}_{g,t}\} \tag{5}$$

for each gene g. This ignores any effect the temporal variation may have had on the observed variance in the data and should overestimate the ω_g.

To estimate the temporal variation, we examine the between time variation in the data. However we need to account for the effect of the covariance function Σ_τ, which models the variation over time. We evaluate Σ_τ at the observed capture times to estimate the covariance of samples from the capture times. This gives a covariance matrix $\hat{\Sigma}$ where

$$\hat{\Sigma}_{t_1,t_2} = \Sigma_\tau(\kappa_{t_1}, \kappa_{t_2}) \tag{6}$$

It can be shown (see Supplementary Material) that a sample from a zero mean Gaussian with covariance matrix Σ is expected to have a variance of

$$V(\Sigma) = \text{Mean}(\text{Diag}(\Sigma)) - \text{Mean}(\Sigma) \tag{7}$$

Using the linearity of this result the expected variance of samples from expression profile y_g at the capture times $\kappa_1, \ldots, \kappa_T$ are expected to have variance equal to

$$\psi_g V(\hat{\Sigma}) + \omega_g \tag{8}$$

We slightly overestimate ψ_g by setting

$$\hat{\psi}_g = \frac{\text{Var}_t(\mathbb{M}_{g,t})}{V(\hat{\Sigma})} \tag{9}$$

where the overestimation is caused by ignoring the effect of ω_g on the expected variance. This should be a small effect as we average over multiple cells per capture time.

Using these estimates for ψ_g and ω_g we empirically set the hyperparameters of the model as

$$\mu_\psi = \text{Mean}\{\log \hat{\psi}_g\} \tag{10}$$
$$\sigma_\psi^2 = \text{Var}\{\log \hat{\psi}_g\} \tag{11}$$
$$\mu_\omega = \text{Mean}\{\log \hat{\omega}_g\} \tag{12}$$
$$\sigma_\omega^2 = \text{Var}\{\log \hat{\omega}_g\} \tag{13}$$

Candidate initialisations

The DeLoren R package offers two separate methods to identify candidate initialisiations for the MCMC chains and ADVI inference.

We sample many (by default, 6000) sets of pseudotimes from the prior and evaluate their likelihood (in combination with empirical Bayes estimates of the other parameters). We choose those samples with the highest likelihoods as the initialisations. We found this naive approach is superior to using random samples from the prior to initialise the chains (data not shown).

The other preferred method is to use the R seriation package to suggest candidate orderings for the cells based on their expression levels. We apply several of the algorithms in the seriation package (TSP, R2E, HC, GW, and OLO by default) to the raw expression data and dimensionality reduced versions of it (we use the PCA, KFA, ICA, and MDS algorithms by default). Again we select those orderings that give the highest likelihoods as candidate orderings.

Algorithm running times

We ran our method on a DELL R815 server with 4 x AMD 6174 2.2 Ghz processors (12 cores each), with 128Gb of 1333 MHz RAM memory (16 x 8192 MB DIMMS). Each initialisation of the ADVI algorithm ran on a separate core concurrently. We present two running times, one to fit the model and also the total required to run the relevant Rmarkdown script that is included with the DeLorean package, including pre- and post-processing the data and saving the results. The low rank model fits approximately 20 times faster on the Shalek data than the exact model on the similarly sized McDavid data.

Data set	genes	cells	model	inference	# inits	fit	total
Windram	100	24	exact	ADVI	40	3m	12m
McDavid	56	361	exact	ADVI	40	7h31m	7h39m
Shalek	74	307	low rank	ADVI	40	20m	42m

Windram Arabidopsis data

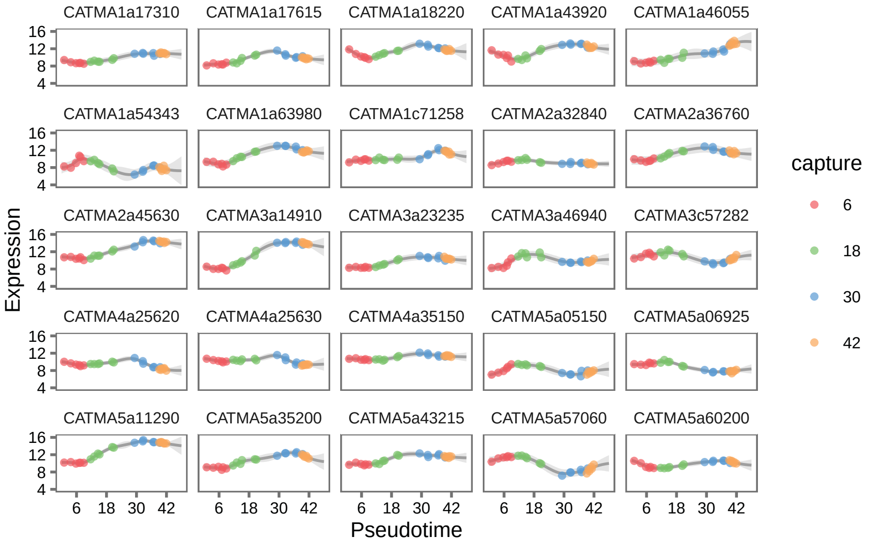

Expression profiles of selected genes from *Arabidopsis* data over pseudotime. The expression data $x'_{g,c}$ are shown as points coloured by their obfuscated capture time. The expected posterior mean of each profile is shown as a grey line and the shaded grey area in each profile represents the posterior uncertainty up to two standard deviations away from the mean.

Monocle analysis

We ran the Monocle algorithm on the same Windram data that we analysed with DeLorean. We did not provide the Monocle algorithm with additional

information such as the root cell and we asked it to fit one path to the whole data set as DeLorean does. The code we used is available in the DeLorean package as an R markdown script: 'inst/scripts/Windram-Monocle.Rmd' and on github: `https://github.com/JohnReid/DeLorean/blob/master/inst/scripts/Windram-Monocle.Rmd`.

Roughnesses

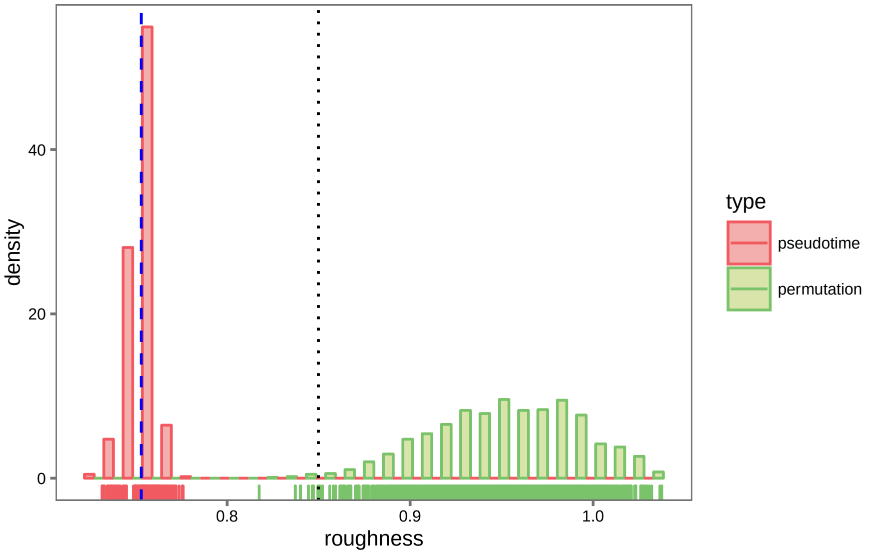

The roughness of the inferred expression profiles for the *Arabidopsis* data Roughnesses of samples from the posterior of our model (red) and from draws from the null hypothesis (green). The roughness of the best sample from our posterior is shown as a dashed blue line. The roughness of the Monocle pseudotime ordering is shown as a dotted line.

McDavid cell cycle data

Oscope analysis

We used Oscope to estimate the pseudotimes. Oscope's primary functionality is to identify pairs and clusters of co-oscillating genes however it does include an extended nearest insertion pseudotime estimation algorithm. Full details of the protocol we followed to run Oscope are in the Rmarkdown script McDavid-Oscope.Rmd included in the DeLorean package but we give an overview here.

We used the same genes as in the DeLorean analysis. In the K-medoids algorithm we used all the pairs of genes as McDavid et al.'s analysis had shown they are all cell cycle genes. We ignored (flagged) one insignificant cluster as per the Oscope method. Once we had a pseudotime ordering from the extended nearest insertion algorithm we needed to align it to the cell cycle. We did this by

minimising the RMSE between the cells' cell cycle phase labels and the centre of the phase in pseudotime.

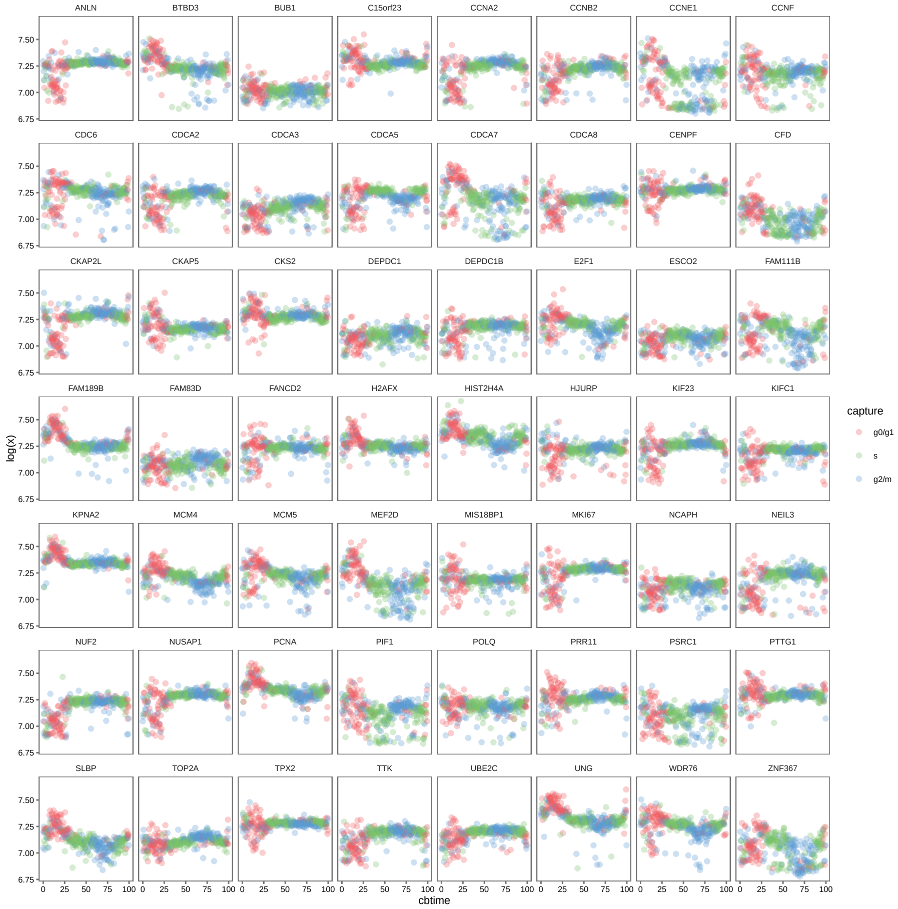

Gene expression profiles inferred by Oscope's extended nearest insertion algorithm. The S phase cells appear to have been split into two groups, one placed before most of the G2/M cells and one after.

Shalek mouse dendritic cells data

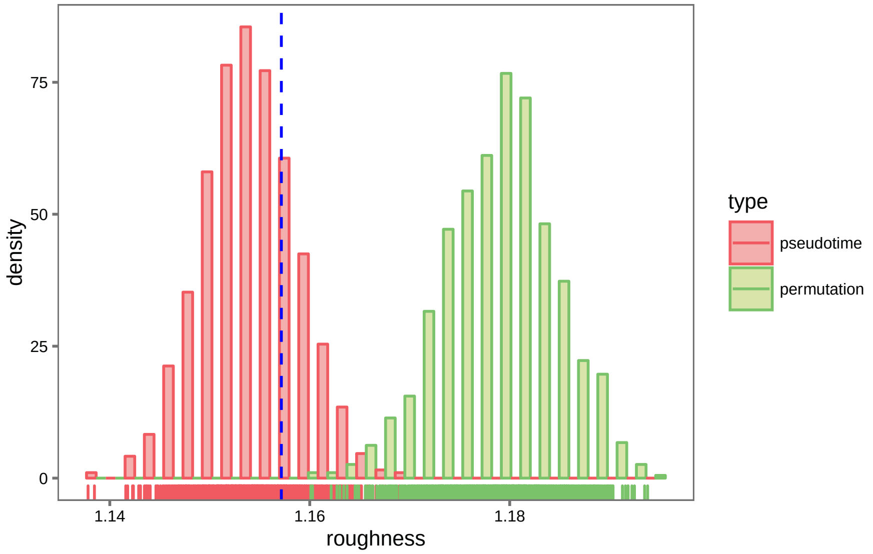

The roughness of the inferred expression profiles for the LPS stimulated mouse dendritic cells Roughnesses of samples from the posterior of our model (red) and from draws from the null hypothesis (green). The roughness of the best sample from our posterior is shown as a dotted blue line.

References

[1] S. Anders and W. Huber. Differential expression analysis for sequence count data. *Genome Biology*, 11(10):R106, Oct. 2010.

[2] J. Quiñonero-Candela and C. E. Rasmussen. A unifying view of sparse approximate Gaussian process regression. *The Journal of Machine Learning Research*, 6:1939–1959, 2005.

[3] E. Snelson and Z. Ghahramani. Sparse Gaussian processes using pseudo-inputs. 2006.
